# Institute of Medicine Forum on Emerging Infections: Linking Infectious Agents and Chronic Diseases

**DOI:** 10.3201/eid0812.020717

**Published:** 2002-12

**Authors:** Siobhán O’Connor, Thomas M. Shinnick, Christopher E. Taylor

**Affiliations:** * Centers for Disease Control and Prevention, Atlanta, Georgia, USA; † National Institutes of Health, Bethesda, Maryland, USA

The belief that many long-recognized chronic diseases are infectious in origin dates to the mid-nineteenth century, when cancer was studied as a possible infectious disease. In the 1950s and 1960s, much biomedical research was unsuccessful in confirming microbial causes of various chronic syndromes. Recent years, however, are marked by successful identification of several causal infectious agents of chronic disease such as *Human papillomavirus* in cervical cancer, but challenges and controversies remain. 

The Institute of Medicine’s Forum on Emerging Infections recently sought to address this rapidly evolving field. To identify cross-disciplinary contributions and challenges in determining infectious causes of chronic diseases, the forum hosted a 2-day workshop on October 21–22, 2002, Linking Infectious Agents and Chronic Diseases: Defining the Relationship, Enhancing the Research, and Mitigating the Effects. In response to invited presentations, participants explored factors driving infectious causes of chronic diseases to prominence, identified difficulties in linking infectious agents with chronic conditions, and discussed broad-based strategies and research programs that might advance the field. 

Invited experts provided research findings on a diverse range of recognized and potential chronic sequelae of infection as well as diverse pathogenic mechanisms from exposure to chronic outcome. Cancers, demyelinating syndromes, cardiovascular disease, neuropsychiatric diseases, hepatitis, and diabetes mellitus were among the chronic conditions addressed. Ensuing discussions noted gaps in knowledge and in the translation of research data to health-care interventions for both accepted and speculative causal associations. Workshop participants remarked on the likely widespread clinical and public health implications of linking infectious agents with chronic diseases that dominate health care in economically established countries, including the United States. The potential benefits of detecting and preventing causal infections, and the risks of interventions against unproven causal agents, are substantial. Workshop participants advocated careful research to produce and appropriately translate validated, reproducible data into clinical management to alleviate the impact of chronic diseases.

Participants also recognized the potential impact of infectious disease control on chronic diseases in economically developing countries. Within 20 years, chronic diseases are expected to represent a substantial proportion of their health burden. Presentations on human T-cell lymphotropic virus type 1 infection and hepatitis C–schistosomiasis coinfection demonstrated the impact of progressive chronic infections that disproportionately affect developing regions. These presentations emphasized the importance of considering coinfections in chronic disease pathology. Data on chronic outcomes of malaria in infected persons and in unborn children and of other coinfections emphasized these points. Presentations also examined causal associations between enteric or parasitic infections and long-term developmental disabilities, as well as links between infectious agents and epilepsy. Coinfections and common acute infections may represent an under-recognized source of chronic pathology. In regions with limited health-care resources, newly identified infectious causes of chronic diseases, including tuberculosis and malaria, may require increased attention.

Against the backdrop of multiple microbes and multiple chronic outcomes, participants attempted to identify research opportunities, challenges, and barriers to understanding linkages between infections and chronic syndromes, and ultimately efforts to mitigate the impact of chronic diseases on human health. Recent developments in technology, methodology, and collaborative research have clearly advanced the ability to determine causal relationships. However, the workshop highlighted numerous factors that complicate identification and confirmation of one or more infectious roots of a chronic disease—factors that current and future research must address. These challenges include possible multifactorial pathogenesis such as interactions between environmental and genetic (host and microbe) influences; how the timing of infection determines final chronic outcome; and the “hit-and-run” nature of certain microbes that may be eliminated before chronic disease becomes apparent. Additional challenges include differentiating the roles of acute, persistently active, latent, and recurrent infection in pathogenesis; the possible singular role of certain species or strains in producing chronic sequelae; the influence of coinfections in defining final pathology; difficulty detecting latent infection before or when chronic disease is diagnosed; differences in the sensitivity and specificity of detection assays in different tissues; difficulty culturing certain microbes; and the lack of adequate methods to identify novel or rare microbes, viruses, and other pathogens. Equally complex is balancing investment in potential infectious causes of multifactorial, high burden diseases with that for rarer conditions that may have one primary cause, infection.

Participants noted that recently developed molecular and immunologic techniques (e.g., representational difference analysis, gene-chip profiling of host and microbe, immune response profiling, proteomics) offer new ways to overcome several obstacles to identifying potential etiologic agents. However, continued investment in new technologies or improving existing methods remains key to overcoming challenges. Defining the temporal relationship between infection and chronic disease with appropriate technology is critical to translating science into effective clinical strategies that intervene against infection to prevent or minimize chronic disease.

Discussion further emphasized that scientifically sound, new technologies must be applied to and guided by a foundation of epidemiologic clues from well-designed studies and surveillance systems. A multipronged approach will be critical. Research and public health activities need to facilitate appropriate linkage of existing and newly designed databases, ensuring quality surveillance and epidemiology that better characterize infectious and chronic diseases with their distributions and potential associations. Many settings demand longitudinal investigations to complement case-control or cross-sectional studies, requiring longer term investment. Detecting and confirming causal associations will require study of both larger cohorts and at-risk subpopulations. 

Improved coordination between basic and clinical scientists, pathologists, and epidemiologists is critical to these goals. Networks and collaborative teams are needed to develop and demand the necessary standardized case definitions (for the infection and the chronic outcome), new and adequate specimen collections with pedigree databases, and comparable methods of analysis. Overall discussions emphasized two major themes of the workshop: 1) the need to define the nature and scope of future research that balances global efforts among the various chronic syndromes and 2) development of a coordinated and systematic strategy to maximize resource use and overcome the inherent technologic, epidemiologic, and organizational challenges in this field.

A published summary of the workshop that includes individually authored papers by workshop presenters will be available in early 2003 from the National Academy Press (available from: URL: www.nap.edu or 800-624-6242). Additional information about the activities of the Forum on Emerging Infections can be found at URL: http://www.iom.edu under the Board on Global on Health or by contacting 202-334-3992. 

